# Emergency palliative cancer care: anxiety and midazolam

**DOI:** 10.1186/s12904-025-01687-5

**Published:** 2025-03-13

**Authors:** Morten Tranung, Tora S. Solheim, Erik Torbjørn Løhre, Morten Thronaes, Michael Due Larsen

**Affiliations:** 1 Department of Clinical and Molecular Medicine, Faculty of Medicine and Health Sciences, Norwegian University of Science and Technology, Trondheim, Norway; 2 Department of Clinical Pharmacy - Trondheim Hospital Pharmacy, Trondheim, Norway; 3 Cancer Clinic, St. Olavs hospital - Trondheim University Hospital, Trondheim, Norway; 4 Centre for Crisis Psychology, Faculty of Psychology, University of Bergen, Bergen, Norway; 5 Centre for Clinical Epidemiology, Odense University Hospital, Odense, Denmark

**Keywords:** Anxiety, Dyspnoea, Pain, Benzodiazepines, Midazolam, Palliative Care, Palliative Medicine

## Abstract

**Background and Objective:**

Cancer patients treated with palliative intent often report anxiety. Anxiety is associated with dyspnoea, cancer pain, and reduced quality of life. Limited knowledge on variability and treatment effects warranted exploring factors associated with improvement in anxiety for hospitalised palliative cancer patients.

**Methods:**

This study is a cross-sectional secondary analysis. All patients admitted to an acute palliative care unit for one year were assessed and 164 patients satisfied the study inclusion criteria. The patients reported self-registered symptom intensities using the 11-point numeric rating scale. Demographic variables, patient reports, and medical management were analysed for associations with anxiety.

**Results:**

At admission, 37.8% of the patients reported moderate or severe anxiety, and of these 43.6% used benzodiazepines. The corresponding numbers for benzodiazepine use were 35.1% and 24.4% for patients with mild and no anxiety, respectively. Of all patients, 26.8% reported improved anxiety during their hospital stay. More patients with moderate or severe anxiety at admission reported improved anxiety during hospitalisation (50.0%) compared to the corresponding patients with mild anxiety (22.8%). Patients with moderate or severe anxiety reported less improvement in pain compared to patients with mild anxiety. Improved dyspnoea was the only factor statistically associated with improvement in anxiety, both for patients reporting mild anxiety and moderate and severe anxiety. Thirty-seven-point-1% of patients with moderate or severe anxiety at admission received no benzodiazepine treatment during the hospital stay. Patients receiving midazolam had more anxiety at admission, were younger, and had poorer performance status. Median dose and interquartile range [IQR] of midazolam in these patients were 2 mg/24 h [IQR: 2.0–6.0].

**Conclusion:**

Improved dyspnoea was associated with reduced anxiety; however, the use of benzodiazepines was not.

## Introduction

Anxiety, pain, and dyspnoea are prevalent symptoms in palliative cancer care, and both cancer pain and dyspnoea may be associated with increased anxiety [1–4]. Studies on symptom clusters in palliative care has also shown that anxiety is interrelated with pain and dyspnoea, as well as other frequently experienced symptoms in palliative care such as tiredness, depression, and somnolence, among others [5]. In addition, anxiety affects quality of life [6, 7]. Thus, anxiety might result in reduced coping and inferior symptom control. There is limited knowledge regarding factors related to anxiety development during hospitalisation, and limited evidence to guide therapeutic interventions [8–10]. In a study on symptom relief in cancer patients during hospitalisation in an acute palliative care unit (APCU), anxiety was the single symptom, next to depression, with the least patient-reported improvement [11].

Non-pharmacological interventions, oral benzodiazepines, and parenteral midazolam are all relevant treatment options for anxiety in palliative care [10]. Despite limited guidance on safety and effectiveness, benzodiazepines like midazolam are widely used in this population [12–14]. Concerns regarding the negative impacts of benzodiazepine consumption can potentially limit their utilization, even though a systematic review found no negative influence on survival for cancer patients using these pharmaceuticals [15, 16]. Benzodiazepines may reduce anxiety and might, co-administered with other medications, play a role in controlling both pain and dyspnoea [12, 17, 18]. Nevertheless, strong opioids represent the mainstay of treatment for moderate to severe cancer-related pain [19]. Opioids also relieve dyspnoea and are recommended in updated guidelines on management of breathlessness in patients with advanced cancer [20, 21]. However, benzodiazepines may be indicated for selected patients with severe anxiety and concomitant dyspnoea [22]. The described anxiety-suppressing potential of opioids further complicates the clinical picture [23].

Separating mild from more severe symptom intensities may facilitate appropriate interventions and advantageous prioritization of health resources [24]. Different views have been advocated with respect to the most relevant cut points between mild and more severe symptom intensities [25]. However, positive scores ≤ 3 on the 11-point numeric rating scale (NRS 0–10) are often considered mild and acceptable, and scores ≥ 4 indicative for follow-up [25–27]. For further categorization, NRS scores 4–6 may be viewed as moderate and scores 7–10 as severe symptom intensities [28, 29].

Anxiety is burdensome and may also accentuate other symptoms. Despite limited guidance, relevant interventions to mitigate anxiety are important in patients with advanced cancer [10, 30, 31]. We aimed to describe anxiety development during hospitalisation in an APCU. Specifically, we addressed the following research questions:

i) What characterized patients admitted with moderate and severe anxiety?
ii) How did anxiety intensity change during hospitalisation for patients with no and mild anxiety at admission?
iii) In which way was anxiety development associated with control of pain, dyspnoea, or the use of specific medications during hospitalisation for patients admitted with moderate and severe anxiety?

## Methods

**Fig. 1:**
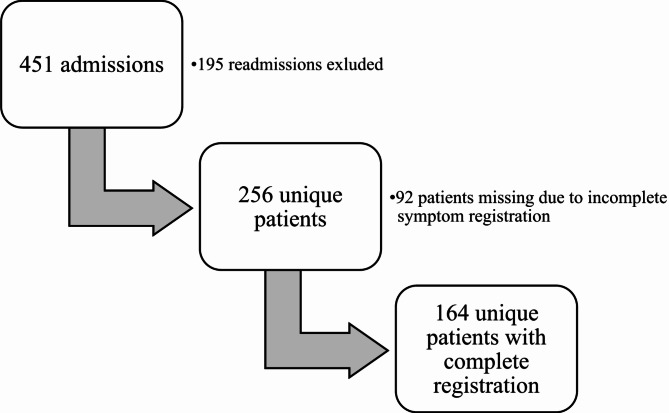
Study population flowchart

This cross-sectional secondary analysis is based on data from a prospective study including all patients hospitalised between January 15, 2019, and January 15, 2020, at the APCU, Cancer Clinic, St. Olavs hospital, Trondheim University Hospital, Norway [11]. The cohort consisted of adults with incurable cancer and referred to palliative care. Both patients with concurrent and no ongoing cancer treatment were included. Patients with haematological, gynaecological, and pulmonary malignancies were included only if they received neuraxial pain management administered by the APCU, as these patients receive care from their respective specialties due to the organisation of the hospital [11]. The current study included all patients with available self-reported NRS 0–10 registrations for anxiety, pain, and dyspnoea both at admission and at discharge (Fig. 1). For patients who died during the hospital stay, last available registrations were applied. Readmissions were excluded.

Patient-reported outcome measures (PROMs) of anxiety, average pain intensity, and dyspnoea at admission and discharge were obtained. For all symptom scores, the assessment period was past 24 h. In addition, physician registered information on patient demographics, including age, gender, marital status, cancer diagnosis, metastatic status, Eastern Cooperative Oncology Group performance status (ECOG PS), if they were receiving integrated cancer care (palliative care and oncological treatment) or palliative care only, and the use of benzodiazepines and opioids was retrieved [32].

The patients were categorized as having no (NRS 0), mild (NRS 1–3) or moderate and severe (NRS 4–10) anxiety [28, 29]. In addition, the patients were grouped as having no (NRS 0), mild (NRS 1–3), moderate (NRS 4–6), or severe (NRS 7–10) pain and dyspnoea, respectively [28, 29]. Furthermore, symptom intensities were considered improved if decreased one point or more, deteriorated if increased one point or more, and otherwise stable [33]. The use and dosage of oral benzodiazepines and parenteral midazolam were assessed both at admission and discharge. Opioid doses were categorized as stable, increased, or decreased during hospitalisation.

Descriptive statistics was used with means and medians as measures of central tendency and standard error of the mean (SEM) and interquartile range (IQR) as measures of dispersion. The Shapiro-Wilk test was used to investigate normal distribution of continuous variables. Mean change in symptom score was calculated for anxiety, pain, and dyspnoea. Univariate and multiple logistic regression analyses were conducted to check if any explanatory variables were associated with improvement in anxiety. Improvement in anxiety was measured as a binary outcome variable, where a positive change in anxiety was measured as improvement, and the remaining as no improvement. The explanatory variables included were age, gender, marital status, pharmacological anxiety treatment (oral benzodiazepines or subcutaneous midazolam), opioid treatment, improvement in pain, and improvement in dyspnoea. A univariate logistic regressions analysis was performed with one explanatory variable at a time to pinpoint factors associated with improvement in anxiety. Similarly, a multiple logistic regression was conducted using all explanatory variables for analysis of the independent effect. Regression models were stratified by anxiety levels at admission (mild and moderate and severe). The statistical analyses were performed using Stata Statistical Software version 18.0 (StataCorp LP, College Station, TX, USA).

## Results

Of 451 patients admitted during the study period, 164 unique patients fulfilled the inclusion criteria (Fig. 1). The median age was 72 years, two-thirds were males and one-third were living alone (Table 1). The most prevalent cancer category was gastrointestinal (GI) cancers. One-third (34.8%) of the patients reported mild anxiety at admission, two thirds (64.6%) were admitted without ongoing benzodiazepine treatment, and one fourth (26.8%) reported improved anxiety during the hospital stay.

**Table 1:** Patients admitted to the acute palliative care unit at the Cancer Clinic, St. Olavs hospital, Trondheim University Hospital during Jan 15th, 2019, and Jan 15th, 2020, according to self-reported anxiety at admission

	No anxiety	Mild anxiety	Moderate and severe anxiety	Total
N (%)	45 (27.4)	57 (34.8)	62 (37.8)	164 (100.0)
Age, median [IQR]	71.0 [66.0–80.0]	71.0 [62.0–78.0]	72.0 [62.0–79.0]	72.0 [62.5–79.0]
Gender, n (%)				
Male	32 (71.1)	32 (56.1)	44 (71.0)	108 (65.9)
Female	13 (28.9)	25 (43.9)	18 (29.0)	56 (34.1)
Social status, n (%)				
Living alone	15 (33.3)	23 (40.4)	20 (32.3)	58 (35.4)
Married/cohabitant	30 (66.7)	34 (59.6)	42 (67.7)	106 (64.6)
Eastern Cooperative Oncology Group Performance status (ECOG PS*), n (%)				
Score 1	4 (8.9)	7 (12.3)	6 (9.7)	17 (10.4)
Score 2	16 (35.6)	25 (43.9)	21 (33.9)	62 (37.8)
Score 3	21 (46.7)	24 (42.1)	31 (50.0)	76 (46.3)
Score 4	4 (8.9)	1 (1.8)	4 (6.5)	9 (5.5)
Cancer type, n (%)				
GI	21 (46.7)	25 (43.9)	27 (44.3)	73 (44.8)
Urologic	7 (15.6)	16 (28.1)	16 (26.2)	39 (23.9)
Breast	3 (6.7)	7 (12.3)	1 (1.6)	11 (6.7)
Lung	0 (0.0)	0 (0.0)	3 (4.9)	3 (1.8)
Head/neck	6 (13.3)	3 (5.3)	2 (3.3)	11 (6.7)
Other	8 (17.8)	6 (10.5)	12 (19.7)	26 (16.0)
Metastases, n (%)				
No	6 (13.3)	7 (12.3)	6 (9.7)	19 (11.6)
Yes	39 (86.7)	50 (87.7)	56 (90.3)	145 (88.4)
Trajectory, n (%)				
Palliative cancer care	29 (64.4)	31 (54.4)	28 (45.2)	88 (53.7)
Integrated cancer care	15 (33.3)	26 (45.6)	34 (54.8)	75 (45.7)
Missing	1 (2.2)	0 (0.0)	0 (0.0)	1 (0.6)
Anxiety treatment at admission, n (%)				
No treatment	34 (75.6)	37 (64.9)	35 (56.5)	106 (64.6)
Oral benzodiazepine	9 (20.0)	16 (28.1)	22 (35.5)	47 (28.7)
Subcutaneous midazolam	2 (4.4)	4 (7.0)	5 (8.1)	11 (6.7)
Pain at admission, n (%)				
No pain	11 (24.4)	5 (8.8)	9 (14.5)	25 (15.2)
Mild pain	12 (26.7)	15 (26.3)	21 (33.9)	48 (29.3)
Moderate pain	15 (33.3)	27 (47.4)	19 (30.6)	61 (37.2)
Severe pain	7 (15.6)	10 (17.5)	13 (21.0)	30 (18.3)
Dyspnoea at admission, n (%)				
No dyspnoea	20 (44.4)	7 (12.3)	13 (21.0)	40 (24.4)
Mild dyspnoea	13 (28.9)	26 (45.6)	12 (19.4)	51 (31.1)
Moderate dyspnoea	7 (15.6)	16 (28.1)	27 (43.5)	50 (30.5)
Severe dyspnoea	5 (11.1)	8 (14.0)	10 (16.1)	23 (14.0)

* Eastern Cooperative Oncology Group performance status

At admission, 37.8% of all patients reported moderate and severe anxiety, and of these more were male (71.0%) and more used benzodiazepines (43.6%) than patients with no or mild anxiety at admission. The patients with moderate or severe anxiety also more frequently still received oncological treatment and reported a higher degree of dyspnoea.

In patients with no reported anxiety at admission, 31 (68.9%) received no benzodiazepine treatment during their hospitalisation (Table 2). A total of eight patients (17.8%) had an increase in their anxiety treatment, of whom one also reported deteriorated anxiety when measured at discharge. Anxiety deteriorated in 7 patients (15.6%) who reported no anxiety at admission.

For the 57 patients reporting mild anxiety at admission, 31 patients (54.4%) received no benzodiazepine treatment, and 13 patients (22.8%) had no change in their anxiolytic treatment during their stay. Patients with mild anxiety reported more improvement in pain than other groups.

**Table 2:** Change in treatment and symptom scores anxiety patients at admission to the APCU

	No anxiety (*n* = 45)	Mild anxiety (*n* = 57)	Moderate and severe anxiety (*n* = 62)
Benzodiazepine treatment during stay, n (%)			
No treatment	31 (68.9)	31 (54.4)	23 (37.1)
Unchanged	6 (13.3)	13 (22.8)	16 (25.8)
Started/increased oral benzodiazepines	5 (11.1)	6 (10.5)	9 (14.5)
Started/increased subcutaneous midazolam	3 (6.7)	7 (12.3)	14 (22.6)
Opioid treatment during stay, n (%)			
No change	22 (48.9)	23 (40.4)	22 (35.5)
Dose increased	19 (42.2)	28 (49.1)	30 (48.4)
Dose decreased	4 (8.9)	6 (10.5)	10 (16.1)
Anxiety development during stay, n (%)			
Deteriorated	7 (15.6)	5 (8.8)	3 (4.8)
Stable	38 (84.4)	39 (68.4)	28 (45.2)
Improved	N/A*	13 (22.8)	31 (50.0)
Pain development during stay, n (%)			
Deteriorated	3 (6.7)	6 (10.5)	7 (11.3)
Stable	29 (64.4)	23 (40.4)	37 (59.7)
Improved	13 (28.9)	28 (49.1)	18 (29.0)
Dyspnoea development during stay, n (%)			
Deteriorated	4 (8.9)	5 (8.8)	3 (4.8)
Stable	28 (62.2)	37 (64.9)	40 (64.5)
Improved	13 (28.9)	15 (26.3)	19 (30.6)

* As no anxiety reported cannot improve during the hospitalisation, there cannot be any patients in this category

Patients with moderate and severe anxiety received more anxiolytic treatment during their hospitalisation (37.1%) compared to patients reporting no and mild anxiety (20.6% %), and 22.6% of patients with moderate and severe anxiety had either midazolam treatment initiated or had their midazolam dose increased during the stay. These patients reported more improvement in anxiety (50.0%) compared to patients with mild anxiety (22.8%), however for pain development, they reported less improvement than patients with mild anxiety (29.0% and 49.1%, respectively). There were no major differences in the development of dyspnoea during hospital stay for patients reporting moderate and severe anxiety at admission compared to those reporting no and mild anxiety.

There was no significant association between age, gender, or marital status for the improvement in anxiety during hospital stay (Table 3). For pharmacological treatment, there were no significant associations between benzodiazepine treatment or opioid treatment for the improvement in anxiety. The only factor associated with an improvement in anxiety during the hospital stay was the improvement of the patient’s dyspnoea. This result was independent of the initial level of anxiety at admission.

**Table 3:** Logistic regression analysis for factors’ association with improvement on anxiety stratified by anxiety at admission

Moderate and severe anxiety at admission (*n* = 62)	Univariate model		Multivariate model	
Factor (n)*	OR (95% CI)	P-value	OR (95% CI)	P-value
Age	1.02(0.98–1.07)	0.322	1.01 (0.95–1.06)	0.812
Gender				
Female (18)	1.49(0.45–4.93)	0.518	2.89 (0.57–14.68)	0.201
Marital status				
Married (42)	Ref.		Ref.	
Living alone (20)	1.85 (0.56–6.06)	0.312	2.36 (0.44–12.74)	0.317
Benzodiazepine treatment during stay				
No treatment (23)	Ref.		Ref.	
Unchanged (16)	0.78 (0.19–3.18)	0.725	0.56 (0.10–3.23)	0.514
Started/increased oral benzodiazepines (9)	1.24 (0.20–7.67)	0.821	1.41 (0.16–12.53)	0.759
Started/increased subcutaneous midazolam (14)	0.26 (0.06–1.08)	0.065	0.24 (0.04–1.46)	0.122
Opioid treatment during stay				
No (22)	Ref.		Ref.	
Dose increased (30)	0.56 (0.17–1.85)	0.343	1.43 (0.26–7.83)	0.677
Dose decreased (10)	0.88 (0.17–4.54)	0.874	1.99 (0.18-22.00)	0.575
Improved analgesia				
No (30)	Ref.		Ref.	
Yes (32)	0.95 (0.33–2.74)	0.931	0.85 (0.21–3.46)	0.816
Improved dyspnoea				
No (30)	Ref.		Ref.	
Yes (32)	4.33 (1.39–13.55)	0.012	6.64 (1.67–26.39)	0.007
**Mild anxiety at admission (** ***n*** ** = 57)**	Univariate model		Multivariate model	
Factor (n)*	OR (95% CI)	P-value	OR (95% CI)	P-value
Age	1.02(0.98–1.07)	0.242	1.06 (1.00-1.14)	0.067
Gender				
Female (25)	0.56(0.19–1.64)	0.292	0.56 (0.12–2.67)	0.469
Marital status				
Married (34)	Ref.		Ref.	
Living alone (23)	0.97 (0.34–2.83)	0.962	1.14 (0.21–6.20)	0.883
Benzodiazepine treatment during stay				
No treatment (31)	Ref.		Ref.	
Unchanged (13)	0.87 (0.23–3.26)	0.831	0.95 (0.13–7.04)	0.960
Started/increased oral benzodiazepines (6)	2.77 (0.44–17.46)	0.278	3.47 (0.38–31.72)	0.271
Started/increased subcutaneous midazolam (7)	1.04 (0.20–5.45)	0.964	4.03 (0.39–41.58)	0.242
Opioid treatment during stay				
No (23)	Ref.		Ref.	
Dose increased (28)	1.17 (0.38–3.59)	0.788	1.17 (0.19–7.20)	0.869
Dose decreased (6)	3.11 (0.47–20.65)	0.240	8.52 (0.76–96.03)	0.083
Improved analgesia				
No (22)	Ref.		Ref.	
Yes (35)	1.65 (0.55–4.93)	0.367	2.49 (0.46–13.49)	0.291
Improved dyspnoea				
No (27)	Ref.		Ref.	
Yes (30)	4.29 (1.39–13.25)	0.012	9.13 (1.89–44.16)	0.006

* Trajectory was also included in the analyses; however, the results are not shown in this table. No statistically significant association was found for this factor

A total of 29 patients (17.7%) received parenteral midazolam treatment during admission (Table 4). The patients who received midazolam more often had moderate or severe anxiety at admission, were younger, and had poorer ECOG PS score compared to patients not receiving midazolam. A larger percent of patients who received midazolam treatment reported an improvement in anxiety compared to patients not treated with midazolam, however less improvement of dyspnoea. The development of pain was similar between the two groups.

**Table 4:** Descriptive characteristics of patients with or without midazolam treatment during hospital stay

	With midazolam treatment (*n* = 29)	Without midazolam treatment (*n* = 135)
Age, median [IQR]	66.0 [58.0–73.0]	73.0 [64.0–80.0]
Gender, n (%)		
Male	19 (65.5%)	89 (65.9%)
Female	10 (34.5%)	46 (34.1%)
Social status, n (%)		
Living alone	7 (24.1%)	51 (37.8%)
Married/cohabitant	22 (75.9%)	84 (62.2%)
ECOG PS		
Score 1	2 (6.9%)	15 (11.1%)
Score 2	9 (31.0%)	53 (39.3%)
Score 3	14 (48.3%)	62 (45.9%)
Score 4	4 (13.8%)	5 (3.7%)
Anxiety at admission		
No anxiety	5 (17.2%)	40 (29.6%)
Mild anxiety	10 (34.5%)	47 (34.8%)
Moderate and severe anxiety	14 (48.3%)	48 (35.6%)
Anxiety development during stay		
Deteriorated	3 (10.3%)	12 (8.9%)
Stable	17 (58.6%)	88 (65.2%)
Improved	9 (31.0%)	35 (25.9%)
Pain development during stay		
Deteriorated	3 (10.3%)	13 (9.6%)
Stable	15 (51.7%)	74 (54.8%)
Improved	11 (37.9%)	48 (35.6%)
Dyspnoea development during stay		
Deteriorated	4 (13.8%)	8 (5.9%)
Stable	20 (69.0%)	85 (63.0%)
Improved	5 (17.2%)	42 (31.1%)
Midazolam dosage increase in mg/24 h, median [IQR]	2.0 [2.0–6.0]	N/A

The median dose of midazolam at admission was 2.0 mg/24 h [IQR: 2.0–4.0], and similar to the median dose increase of midazolam during the stay. The median dose of midazolam at discharge was also 2.0 mg/24 h [IQR: 2.0–6.0]. In two patients, midazolam treatment was discontinued, and in two patients the dose was reduced during the stay. The maximum administered midazolam dose registered during the study was 50 mg/24 h.

## Discussion

The level of patients’ anxiety observed in our study was comparable to previous studies [1, 2]. There were no significant differences in anxiety observed between age, gender, and social status at admission. Improvement in dyspnoea during the hospital stay was the only statistically significant factor associated with improvement in anxiety. The group of patients who were receiving midazolam were younger, had poorer ECOG PS score, and were more inclined to deteriorated dyspnoea during the stay.

About 30% of patients in palliative cancer care experience anxiety [1, 2]. In our study almost 4 of 10 patients reported a clinically relevant anxiety score (NRS > 3), which is comparable to the aforementioned studies.

The use of benzodiazepines for anxiety is widespread, discussed, and not necessarily founded on solid scientific evidence [10, 34, 35]. A survey conducted in the United Kingdom reported that about 9 out of 10 patients diagnosed with anxiety who had a shorter prognosis (days-weeks) were prescribed benzodiazepines, and among patients with a longer prognosis (months) about 5 in 10 received benzodiazepines [8]. In the present study, 6 out of 10 patients with moderate or severe anxiety received benzodiazepine treatment during the hospital stay. The studies are not directly comparable as our study has not differentiated on prognosis, survival or for how long patients have been treated with benzodiazepines, and as such, we cannot say if this is regarded as short-term or long-term use of benzodiazepines. Based on the limited evidence regarding the effect of benzodiazepines, it is difficult to ascertain whether this represents overuse of benzodiazepines resulting in side effects and lack of potential benefits of dose increases, or if there is an underconsumption of medication and that anxiety is under-focused at the APCU. However, it is certain that almost 4 out of 10 patients who reported moderate or severe anxiety at admission received no pharmacological anxiolytic treatment, and that there is a need for the development of better pharmacological options both for short-term and long-term treatment.

Cochrane reviews have pointed out a lack of evidence for the effectiveness of benzodiazepines for the treatment of anxiety in palliative care, and in our study, the use of benzodiazepines as well as opioids were not significantly associated with improvement in anxiety [10]. The design of the study, and the size of treatment groups, makes it difficult to draw any unambiguous conclusions as to the impact of these pharmacological treatments in palliative care. However, when all benzodiazepine treatment were pooled together, no statistically significant association was seen on improvement in anxiety. This raises questions on both benzodiazepine efficacy and dosing, such as dose threshold where an increase in dose to achieve symptom management can lead to severe side effects. Additionally, the development of patients’ anxiety intensity without the use of benzodiazepines is unknown.

Previous studies in palliative care have shown that patients with a high degree of anxiety have a reduced tolerance of pain and report a higher frequency of pain [30, 36]. This can explain why our patients with moderate or severe anxiety at admission had less improvement in pain than patients with no or mild anxiety. The pathophysiology behind this association is not clearly elucidated, however it emphasises the complexity of pain treatment, where concomitant treatment of other symptoms such as anxiety, can potentially help in reducing the perception of pain.

As previous studies on symptom clusters in palliative care have shown, anxiety is interrelated to several other symptoms such as depression, tiredness, and lack of well-being among others [5, 37, 38]. Studies on symptoms in palliative care report that depressive mood and depression are common symptoms experienced by palliative cancer care patients [1, 2]. As one of the research questions were to investigate the effectiveness of midazolam in palliative care, we chose dyspnoea and pain as exploratory symptoms as midazolam have been reported used for these symptoms by healthcare professionals [18]. Studies on the co-existence of anxiety and depression, and further the effects of antidepressant medications on anxiety, in palliative care could yield new treatment strategies.

Integrating palliative care in early disease trajectory has shown promising effects on patients’ quality of life [39, 40]. Interestingly, we found that there was a higher proportion of patients receiving integrated cancer care that reported moderate or severe anxiety at admission, which is in contrast to other findings where anxiety is similar between patients with early palliative care and standard care [41]. The reason for this is unclear, however patients still on active cancer treatment may to a higher degree regard being admitted to the APCU as a sign of disease progression and may to a less extent have processed this disconcerting situation, and regard hospitalisation as a deterioration of their disease. This can be associated with cancer treatment more directly [42], or be linked to uncertainty which has been shown to affect quality of life in patients with advanced cancer [43].

A study in outpatient palliative care showed that patients with mild anxiety at baseline tend to have their anxiety deteriorate over time [29]. This can be due to a general progression of disease, or a worsening of just anxiety itself. We observed in our study that one of six patients who did not report any anxiety at admission experienced a worsening of anxiety, which reminds us that anxiety experience is dynamic and should not be omitted even though patients at a single point in time report a lack of it. Additionally, it emphasises the use of validated tools such as ESAS to both capture improvement in PROMs after therapy has been initiated, but also to capture progression of symptoms that were not present at a given time. Anxiety may also be given lower priority in favour of other symptoms more readily observed such as nausea, constipation, and pain, as shown in a Danish study including patients with advanced cancer [44].

The relationship between experiencing dyspnoea and anxiety is well-known, and studies have shown that a worsening of anxiety symptoms were associated with dyspnoea [45, 46]. Given this relationship, reducing the symptom burden of one could potentially reduce the intensity of the other, and studies have shown that improving anxiety can improve dyspnoea [47–49]. We observed an association between improving dyspnoea and concurrent improved anxiety in our study, which underlines that these symptoms are affected by one another. As such, treatment strategy for either symptom should include evaluating and, if needed, treatment of the corresponding symptom.

Previous studies on anxiety in palliative care have shown that introducing integrated palliative care and implementation of international guidelines reduce anxiety [50, 51]. These studies indicate that the largest effect on reducing anxiety in palliative care is not comprised of single factors, but the palliative care approach as a whole. As such it can be difficult to find an association between improvement in anxiety and small-scale interventions. In our study, receiving integrated palliative care was not independently associated with an improvement in anxiety, however it was not recorded when patients were referred to palliative care. Given this, patients could have been receiving palliative care for a longer period of time, and as such we cannot conclude if introducing palliative care improves anxiety or not.

The effects of interdisciplinary treatment approaches in palliative care have been studied, where studies have shown that palliative care patients have improved quality of life, as well as improvement in symptoms such as anxiety and depression [52, 53]. Since studies on pharmacological approaches for anxiety treatment in palliative care have yet to show effectiveness, implementing interdisciplinary treatment strategies can be a different approach to helping palliative care patients with anxiety.

A previous study showed that older patients experience less anxiety than younger patients, and as such younger patients may express more distress that instigates midazolam treatment compared to older patients [54]. In addition, a correlation between higher symptom burden and a poorer EGOC PS score, and as such poorer prognosis, was found by another study [55]. This is in conjunction with our study, where patients who were treated with midazolam were younger and had a poorer EGOC PS score compared to the rest of our study population. Lastly, midazolam, up until recently, has been the viable parenteral benzodiazepine option in Norway.

## Strengths and limitations

The strength of this study is the relatively large patient population, which was included unselected and consecutively over a year. In addition, the patient reported anxiety scores were measured using a validated tool [56, 57].

No subsequent admissions for patients during the study period were included in this analysis, which could affect the degree of anxiety at admission. There is a possibility that reported anxiety at subsequent admissions could be higher, as repeated admissions and patients being late in their disease trajectory could imply increase symptom burden. However, including subsequent admissions would introduce bias and dependency in the data, and as such were excluded.

Furthermore, as expected in a frail patient population, there was missing data due to incomplete symptom registration. The reason for incomplete symptom registration might be progressed disease and inability to report PROMs.

A previous study has showed that ESAS performed poorly to screen for depression in palliative cancer care patients [58], while others found that ESAS is a valid tool for screening of depression compared to the Hospital Anxiety and Depression Scale [56]. This raises the question whether the ESAS form or the NRS is appropriate for screening of psychological symptoms in palliative care, and if other tools such as the Patient Health Questionnaire-9 should be used instead to diagnose and warrant further treatment of these symptoms. As such we have focused our attention to pain and dyspnoea as exploratory factors in this study.

The results of this study must be interpreted with caution as retrospective analyses of observational studies are susceptible to confounding and other biases, and are unable to demonstrate causality [59]. Confounding by indication is an issue in this type of study as palliative care is an end-of-life situation where symptoms deteriorate over time, and treatment is initiated in those patients experiencing symptoms [60]. This can make pharmacological treatment, and other interventions look unfavourable as treatment is initiated in more frail patients who are experiencing a higher symptom burden, and where improvement can be difficult because of the underlying situation of end-of-life care. Indications for pharmacological anxiolytic treatments were not recorded. Thus, the drugs might also be used for treatment of other symptoms [18]. Additionally, there were no non-pharmacological treatment of anxiety measured in this study.

## Conclusion

Anxiety was a prevalently reported symptom in our study, and control of dyspnoea was found to be associated with reduced anxiety. Patients receiving midazolam were younger, they reported more intense anxiety at admission and had poorer ECOG PS status than the elderly patients. The use of benzodiazepine and midazolam was independently not associated with improvement in anxiety.

## Data Availability

The dataset used and analysed during the current study are available from the corresponding author on reasonable request.

## Abbreviations

APCU: Acute palliative care unit
NRS 0-10: 11-point numeric rating scale
PROM: Patient–reported outcome measure
ECOG PS: Eastern Cooperative Oncology Group Performance status
SEM: Standard error of the mean
IQR: Interquartile range
